# Biotechnological Perspectives of Omics and Genetic Engineering Methods in Alfalfa

**DOI:** 10.3389/fpls.2020.00592

**Published:** 2020-05-21

**Authors:** Miroslava Hrbáčková, Petr Dvořák, Tomáš Takáč, Michaela Tichá, Ivan Luptovčiak, Olga Šamajová, Miroslav Ovečka, Jozef Šamaj

**Affiliations:** Department of Cell Biology, Centre of the Region Haná for Biotechnological and Agricultural Research, Faculty of Science, Palacký University Olomouc, Olomouc, Czechia

**Keywords:** alfalfa, *Medicago sativa*, genomics, metabolomics, proteomics, stress resistance genes

## Abstract

For several decades, researchers are working to develop improved major crops with better adaptability and tolerance to environmental stresses. Forage legumes have been widely spread in the world due to their great ecological and economic values. Abiotic and biotic stresses are main factors limiting legume production, however, alfalfa (*Medicago sativa* L.) shows relatively high level of tolerance to drought and salt stress. Efforts focused on alfalfa improvements have led to the release of cultivars with new traits of agronomic importance such as high yield, better stress tolerance or forage quality. Alfalfa has very high nutritional value due to its efficient symbiotic association with nitrogen-fixing bacteria, while deep root system can help to prevent soil water loss in dry lands. The use of modern biotechnology tools is challenging in alfalfa since full genome, unlike to its close relative barrel medic (*Medicago truncatula* Gaertn.), was not released yet. Identification, isolation, and improvement of genes involved in abiotic or biotic stress response significantly contributed to the progress of our understanding how crop plants cope with these environmental challenges. In this review, we provide an overview of the progress that has been made in high-throughput sequencing, characterization of genes for abiotic or biotic stress tolerance, gene editing, as well as proteomic and metabolomics techniques bearing biotechnological potential for alfalfa improvement.

## Introduction

Legumes are important food crops for the exponentially growing population, owing to their micronutrient, macronutrient, and secondary metabolite content (Le et al., 2007). Some of these organic compounds (e.g., phytoalexins and chitinases) play roles in plant defense against pathogens and pests (He and Dixon, 2000). Moreover, Fabaceae is one of the most studied plant families, and it has gained high agricultural importance, especially owing to its ability to fix nitrogen in symbiosis with rhizobia (Doyle and Luckow, 2003).

*Medicago sativa* L., commonly known as alfalfa or “lucerne,” belongs to Fabaceae, and its first cultivated form most likely originates from western Persia. It then spread to many regions in Asia, Europe, and America. In addition, Rashmi et al. (1997) and Samac and Temple (2004) reported that alfalfa ranks fourth in terms of acreage and economic value, following corn, soybean, and wheat.

The genus *Medicago* includes both perennial and annual species. Alfalfa is a highly valuable perennial deep-rooted forage legume, especially because of its widespread production, soil protection, and ability to improve nitrogen-limited soils (Radović et al., 2009). It is also widely cultivated for livestock feed (Flajoulot et al., 2005), and is used as a biofuel feedstock for ethanol production, either as hay or silage (McCoy and Bingham, 1988). The biological and agronomical potential of alfalfa, like all other members of the whole legume family, is extraordinary because it requires little to no nitrogen fertilizer for optimal growth (Ebert, 2007). In addition, alfalfa plays an important role as a free fertilizer providing nitrogen to subsequent crops (Triboi and Triboi-Blondel, 2014).

Alfalfa shows a high content of proteins, enzymes (amylase, coagulase, peroxidase, erepsin, lipase, invertase, and pectinase), antioxidants, minerals, and vitamins A, C, K, and E, as well as valuable phytopharmaceutical components (Bora and Sharma, 2011 and references therein). Moreover, alfalfa and some other species of Fabaceae family possess two different thiol redox compounds, namely glutathione (GSH) and the homoglutathione (hGSH), with higher content of hGSH (Klapheck, 1988; Baldacci-Cresp et al., 2012). More specifically, alfalfa shows different ratios of hGSH/GSH in diverse organs such as leaves, stems, and roots (Pasternak et al., 2014). Thus, alfalfa represents one of the most valuable and important forage crops, and can also be used in grasslands as a cover crop for improved weed control. Finally, alfalfa is also suitable for use in the production of recombinant pharmaceutical proteins (Fu et al., 2015) and in phytoremediation (Nirola et al., 2016).

The tetraploid genome of alfalfa and outbreeding mating systems have made selective breeding harder (Zhou et al., 2011; Annicchiarico et al., 2015). Advanced methods such as genomic, proteomic, and metabolomic approaches, as well as gene editing, could lead to the practical applications of genes that have biotechnological value for alfalfa improvement, especially if applied in an integrated and targeted manner. As a result, single or multiple genes might show desirable effects on several agronomically important alfalfa traits, which can significantly accelerate research in comparison to conventional breeding (Singer et al., 2018). Alfalfa is a major source of proteins in the livestock and dairy industries. In the last years, alfalfa production has been displaced to saline environments by major cereals. Therefore, the incorporation of transgenic traits into alfalfa with varying degrees of tolerance to salinity has been developed and this robust approach can improve the productivity and quality of nitrogen-fixing crops (Kang et al., 2016; Stritzler et al., 2018). Genetically engineered glyphosate-resistant alfalfa was commercialized in the United States in 2010. Another alfalfa variety with reduced lignin content stacked to glyphosate resistance trait has been available since 2015. Reduced lignin content in forage legumes can improve their digestibility by animals, thus it is an important forage quality trait (Li et al., 2016; Barros et al., 2019).

The purpose of this review is to provide a perspective on the current state of alfalfa biotechnology research. It focuses mainly on the biotechnological potential of genomic and transcriptomic approaches, biotechnologically valuable genes, gene editing, proteomics, and metabolomics. When appropriate it is compared to barrel medic.

## Genomic Approaches

The identification of genes that affect legume crop production represents an important aim of current genomic studies (Bevan et al., 2017), and this requires knowledge of their full genomic sequences. Technologies for sequencing DNA and RNA have undergone revolutionary improvements (Ari and Arikan, 2016). It is known that after the evolutionary split between monocots and eudicots, several whole genome duplications and triplications had occurred in legumes (Severin et al., 2011; Masonbrink et al., 2017), which might delay whole genome sequencing efforts. The major strength of next-generation sequencing (NGS) is its ability to detect abnormalities across the entire genome. NGS is less costly and has a faster turnaround time compared to classical sequencing methods. New NGS platforms, such as the Roche/454 system (Margulies et al., 2005), Illumina platform (Wang et al., 2012), real-time DNA sequencing by Pacific Biosciences (Eid et al., 2009), Oxford Nanopore system (Lu et al., 2016), and Ion Torrent system (Rothberg et al., 2011), were used for sequencing crop and legume genomes. They have had a major impact on plant research, since they enable the understanding of genomic complexity as well as the identification of genomic variations, such as single nucleotide polymorphisms (SNPs) or insertions/deletions (INDELs; Valliyodan et al., 2017; Abdelrahman et al., 2018). NGS and bioinformatics approaches for high-throughput data analysis are major tools in modern plant breeding programs (Abdelrahman et al., 2015, 2017a,b; Pavlovich, 2017). These modern technologies are also used in legume research, and several recent studies have been devoted to alfalfa genomics using high-throughput genome sequencing (reviewed by Hawkins and Yu, 2018).

### High-Throughput NGS in Genomics and Transcriptomics

Genome sequencing and assembly have been applied to many plant species, including crops. Such genome assemblies serve as common references for alignment with re-sequenced plants (Huang et al., 2012; Schreiber et al., 2018). Large-scale systematic genome sequencing has been carried out in leguminous plants such as *Lotus japonicus* (Sato et al., 2008), *M. truncatula* (release 3.0)^1^, and *Glycine max* (Schmutz et al., 2010). The genome sequence of alfalfa has not yet been published, and current transcriptomic studies and SNP discoveries rely on the barrel medic genome sequence alignment (genome version^2^ *Mt4.*0v1; Young et al., 2011; Tang et al., 2014). Currently, the most advanced genome sequencing method is NGS. It has become the major tool for the development of new molecular markers and for gene identification (Edwards and Batley, 2010). Together with the rapid development of NGS, the number of plants with completely sequenced genomes has dramatically increased (Van et al., 2013; Le Nguyen et al., 2018; Kersey, 2019). Advantages of NGS include lower costs and shorter time requirements. The development of NGS technology contributed to the identification of new genes that had evolved by whole-genome duplication and structural variations in chromosomes (Barabaschi et al., 2012; Van et al., 2013). Reference genome sequences of several legume and crop species are now available, and candidate genes of important SNPs can be rapidly and easily identified (Gao et al., 2012; Van et al., 2013; Le Nguyen et al., 2018; Scheben et al., 2019). Alfalfa is an outbred, tetrasomic tetraploid (2*n* = 4*x* = 32) with eight basic chromosomes and a genome size of 800–1000 Mbp (Blondon et al., 1994). Genetic and genomic resources have been widely explored and developed, but in the absence of a fully sequenced and assembled reference genome for alfalfa, genome of closely related barrel medic is used as a model organism (Zhou et al., 2011). Barrel medic is a diploid species (2*n* = 2*x* = 16) with smaller genome (about 550 Mbp; Piano and Pecetti, 2010). NGS technologies could speed up the discovery of quantitative trait loci (QTLs) and candidate SNPs, which represent common sequence variations among plants and are functionally important. Numerous molecular markers are used in high-throughput genotyping by sequencing (GBS) platforms associated with alfalfa mapping (Hawkins and Yu, 2018), population diversity studies (Herrmann et al., 2018), and genomic selection (Annicchiarico et al., 2016). In the past years, low density linkage maps were constructed on diploid alfalfa (Brummer et al., 1993; Kiss et al., 1993; Echt et al., 1994; Julier et al., 2003). Although several genetic linkage maps have been constructed for tetraploid alfalfa, most of them were framework maps with only few markers (Brouwer and Osborn, 1999; Julier et al., 2003; Musial et al., 2007; Robins et al., 2007; Khu et al., 2013). Li X. et al. (2014) have constructed a saturated genetic linkage map of autotetraploid alfalfa by using GBS. They have shown high synteny between linkage groups of alfalfa and barrel medic, and clearly identified translocations between chromosomes 4 and 8, and small inversion on chromosome 1. The high-density linkage maps contained 3,591 SNP markers on 64 linkage groups across both maternal and paternal genomes of an autotetraploid alfalfa F_1_ population (Li X. et al., 2014).

Genome-wide associated studies (GWAS) are a modern and powerful strategy that can be used to overcome the limitations of conventional QTL mapping. GWAS map genetic loci in a breeding population, relying on linkage disequilibrium (LD; Liu X. P. et al., 2019). Recently, GWAS have been used in the identification of genetic loci in crop species such as soybean (Hwang et al., 2014), maize (Olukolu et al., 2016), barrel medic (Kang et al., 2015), and alfalfa. Zhang T. et al. (2015) evaluated two important features associated with drought resistance, namely drought resistance index (DRI) and relative leaf water content (RWC) under greenhouse conditions in 198 alfalfa cultivars and landraces. These results were then correlated with genomic data obtained through GBS. Subsequent to the QTL mapping approach, GWAS provided identification of 15 loci associated with DRI and RWC. Markers associated with DRI are located at all chromosomes, whereas markers associated with RWC are located at chromosomes 1, 2, 3, 4, 5, 6, and 7. Co-localization of markers for DRI and WRC were found on chromosomes 3, 5, and 7 (Zhang T. et al., 2015). A GWAS approach using more than 15,000 genome-wide SNPs obtained through GBS was applied to examine forage yield and nutritive value-related traits. Five genes, containing known SNPs aligned to the barrel medic genome, were found as candidates in determining fall dry matter yield (*TUBBY-LIKE PROTEIN*), summer dry matter yield (*E3 SUMO-PROTEIN LIGASE SIZ1, RNA-DEPENDENT RNA POLYMERASE FAMILY PROTEIN*), fall stem weight (*UBIQUITIN-LIKE-SPECIFIC PROTEASE ESD4-LIKE PROTEIN*), and cell wall biogenesis (*NUCLEOTIDE-DIPHOSPHO-SUGAR TRANSFERASE FAMILY PROTEIN*; Sakiroglu and Brummer, 2017). Aiming to find markers for alfalfa forage quality, 154 plants originating from the second generation prepared by the outcrossing of three alfalfa cultivars were subjected to GBS, while their half-sib progenies were phenotyped for forage quality parameters under three different growing conditions. Subsequently, GWAS of SNPs was carried out using barrel medic as a reference genome, confirming a polygenic control of quality traits and indicating a substantially different genetic control of a given trait in stems and leaves (Biazzi et al., 2017). Important alfalfa loci for salt tolerance during germination were identified by similar marker-trait association using a GWAS approach (Yu et al., 2016). Remarkably, they used 198 different accessions with potential drought tolerance, whereas DNA libraries were sequenced in two lanes of an Illumina Hi-Seq2000 instrument. Identified SNP markers were located on all chromosomes, with the exception of chromosome 3. Several alfalfa loci showed similar genetic locations to the reported QTLs associated with salt tolerance in barrel medic. The results suggest the similarity of mechanisms controlling salt stress responses in these two species. This study resulted in the identification of 14 genes connected to 23 markers associated with salt tolerance during germination. These include *PEROXYGENASE, B3 DNA-BINDING PROTEIN*, and *CPR5 PROTEIN*, which are linked to cuticle wax biosynthesis and ABA signaling (Yu et al., 2016).

Over the last two decades, several methods have been developed that allowed the examination of global transcriptional changes. The most used ones are the hybridization of cDNAs (DNA microarrays) and the deep sequencing of cDNA (RNA-Seq; Schena et al., 1995; Wang et al., 2009; Lardi and Pessi, 2018). RNA-Seq, a massive parallel sequencing method for transcriptome analysis, was developed 10 years ago (Wang et al., 2009). Transcriptomic studies analyze only the transcribed portion of the genome and provides in-depth sequencing coverage and additional qualitative information such as isoform-specific expression (Abdelrahman et al., 2018). In contrast to microarrays, ribosomal RNA (rRNA) does not hybridize to the chip, as homologous probes are not present. In RNA-Seq, the abundant rRNA is removed (Lardi and Pessi, 2018). Originally, transcriptomic studies were based on Sanger sequencing of expressed sequence tags (ESTs) or microarrays, which was used in alfalfa and barrel medic (Aziz et al., 2005; Cheung et al., 2006; Yang et al., 2010). It has also been applied for other legumes such as *G. max* (Le et al., 2012; Ha et al., 2015; Tripathi et al., 2016), *L. japonicus* (Asamizu et al., 2004), and *Cicer arietinum* (Deokar et al., 2011).

Several studies contributed to the transcriptome sequencing of alfalfa with various coverage. These studies relied on NGS technologies such as 454 technology (Han et al., 2011) or RNA-Seq (Yang et al., 2011; Li and Brummer, 2012; Liu et al., 2013; O’Rourke et al., 2015). Liu et al. (2013) performed *de novo* transcriptome sequencing of *M. sativa* L. subsp. *sativa* using Illumina paired-end sequencing. Plant material included 15 tissue types, and the transcriptome coverage was 5.64 Gbp of clean nucleotides. About 40,433 unigenes were obtained, and 1649 potential expressed sequence tags simple sequence repeat markers (EST-SSRs) were annotated by alignment with the following databases: the National Center for Biotechnology Information (NCBI) nonredundant protein (Nr) database, the NCBI non-redundant nucleotide sequence (Nt) database, Swiss-Prot, The Kyoto Encyclopedia of Genes and Genomes (KEGG), the Clusters of Orthologous Group (COG), Translated EMBL (TrEMBL), and the InterPro (Ipr) database (Liu et al., 2013). RNA-Seq analysis of two alfalfa subspecies, namely *M. sativa* ssp. *sativa* (B47) and *M. sativa* ssp. *falcata* (F56) using roots, nitrogen-fixing root nodules, leaves, flowers, elongating stem internodes, and post-elongation stem internodes resulted in 112,626 unique transcript sequences, which were assembled into the alfalfa Gene Index 1.2 (MSGI 1.2; O’Rourke et al., 2015). Chao et al. (2019) used PacBio SMRT technology and identified 72,606 open reading frames (ORFs) including 46,616 full-length ORFs, 1670 transcription factors and 44,040 SSRs. A total of 7568 alternative splicing events and 17,740 long non-coding RNAs supported the feasibility of deep sequencing full length RNA from alfalfa transcriptome on a single-molecule level (Chao et al., 2019). Another approach developed to provide long-read sequencing of transcripts is Oxford Nanopore Technologies^®^. The MinION device, which was developed by Oxford Nanopore, is a portable apparatus compatible with a PC or laptop (Jain et al., 2016; Lu et al., 2016). Fleming et al. (2018) evaluated changes in mRNA in dry soybean seeds with use of MinION-based pipeline technology. Li et al. (2019) used MinION-based technology for high-throughput mapping of transgenic alleles in soybean. They rapidly mapped the transgene insertion positions in 51 transgenic soybean plants in a single 1D sequencing run. This method was optimized using a population of soybean lines, but it can be adapted to map the transgenes in any other crops.

## Transcriptomic Approaches and Gene Expression Modifications

### Resistance to Abiotic Stress

Salinity stress interferes with plant growth because it causes two main stresses on plants: hyperosmotic pressure and ion toxicity, especially due to Na^+^ (Volkov et al., 2004). High salinity often triggers an increase in cytosolic Ca^2+^, reactive oxygen species (ROS), abscisic acid (ABA), and mitogen activated protein kinase (MAPK) signaling (Ovečka et al., 2014; Mittler and Blumwald, 2015). These activated signal molecules affect plant transcriptomes by regulating transcription factors (Xiong et al., 2002; Zhu, 2002). One of the basic strategies in plant stress responses is the accumulation of water-soluble compounds of low molecular weight, such as betaines, polyols, sugars, and amino acids (Chen and Murata, 2002). These compounds accumulate to high concentrations under water or salt stress and protect plants via ROS detoxification and membrane integrity maintenance (Bohnert and Jensen, 1996). For example, glycinebetaine (GB) is a particularly effective protectant against abiotic stress (Chen and Murata, 2008), and accumulates rapidly in plants exposed to salt, drought, and low temperature stresses (Rhodes and Hanson, 1993).

Previous studies have shown that overexpression of stress-related genes caused enhanced tolerance of alfalfa to the salinity stress (Luo et al., 2019b). Li H. et al. (2014) successfully targeted *CHOLINE OXIDASE A* (*CODA)* cDNA derived from *Agrobacterium globiformis* to alfalfa chloroplasts under the control of the strong stress inducible *SWEETPOTATO PEROXIDASE ANIONIC 2* (*SWPA2*) promoter (Kim et al., 2003). Such transgenic alfalfa plants exhibited increased tolerance to oxidative, drought, and salt stress. Because salinity also causes cellular ionic imbalances, the Na^+^/H^+^ antiporter in the plasma membrane (SOS1 – SALT OVERLAY SENSITIVE 1) and tonoplast (NHX2 – SODIUM/HYDROGEN EXCHANGER 2) can maintain higher K^+^/Na^+^ ratios in the cytoplasm as a protection against sodium toxicity (Fukuda et al., 1999; Xia et al., 2002; Zhang L. Q. et al., 2014). Moreover, the expression of foreign genes, such as *TaNHX2* (*Triticum aestivum NHX2*), *AhBADH* (*Atriplex hortensis BETAINE ALDEHYDE DEHYDROGENASE*), *SsNHX1 (Suaeda salsa NHX1)*, and *GmDREB1* (*G. max DEHYDRATION-RESPONSIVE ELEMENT BINDING PROTEIN 1*), can increase salt tolerance in transgenic alfalfa plants (Zhang et al., 2012). As such, Zhang L. Q. et al. (2014) transformed the exogenous gene *SeNHX1* (*Salicornia europaea NHX1*) into alfalfa using *Agrobacterium-*mediated transformation; this enhanced tolerance to salt stress was manifested by improved photosynthesis and membrane stability. Another attempt to improve salt tolerance in alfalfa was reported by Jin et al. (2010) using transformation with the soybean *DREB* ortholog, *GmDREB1*, under the control of *Arabidopsis* stress-inducible *RD29A* (*RD – RESPONSIVE TO DESICCATION)* promoter. Ion leakage, chlorophyll fluorescence, total soluble sugars, transcript level of Δ*1-PYRROLINE-5-CARBOXYLATE SYNTHASE* (*P5CS*), and free proline contents were correlated with the higher salt tolerance of transgenic lines (Jin et al., 2010). Wang et al. (2014) generated and characterized transgenic alfalfa plants with heterologous expression of *AtNDPK2* (*NUCLEOSIDE DIPHOSPHATE KINASE 2*) under the control of oxidative stress inducible *SWPA2* promoter. These transgenic plants showed increased tolerance to oxidative, high temperature, salt and drought stresses. Such enhanced tolerance was mediated by activation of ROS scavenging, enhanced activity of NDPK2 enzyme, improved protection of membrane integrity, and increased proline accumulation (Wang et al., 2014).

First studies on drought responses of alfalfa started in the 1990s (Luo et al., 1991, 1992; Laberge et al., 1993). Metabolite profiling and proteomic approaches identified soluble sugars, amino acids, and proteins that respond to drought in leaves and nodules of alfalfa variety Magali (Aranjuelo et al., 2011). Simultaneously, Kang et al. (2011) have shown systematic analysis of two alfalfa varieties, Wisfal and Chilean, with different tolerance/sensitivity to the drought stress. They have identified many genes involved in adaptation to the drought stress, including genes encoding transcription and regulatory factors, or genes involved in the biosynthesis of osmolytes and antioxidants. Knowledge of such genes can help in breeding programs. A number of microRNAs have been used to improve various crop species via genetic engineering (Macovei et al., 2012; Zhou and Luo, 2013; Aung et al., 2015). Researchers also characterized microRNAs and their target genes that respond to hypoxia, wounding, heat or oxidative stress (Zhao et al., 2007; Budak et al., 2015). Recent study by Arshad et al. (2017) suggested that overexpression of microRNA156 (*miR156OE*) is an emerging tool to improve drought tolerance of alfalfa since it silenced *SQUAMOSA PROMOTER BINDING PROTEIN-LIKE 13 (SPL13i)* leading to reduced water loss and enhanced stomatal conductance and photosynthetic assimilation. Another study proposed a role of *miR156OE* and *SPL13i* in heat stress tolerance since plants carrying these constructs showed increased antioxidant levels (Matthews et al., 2019). As found by NGS, plants possessing *miR156OE* exhibited broad changes in gene expression, including genes involved in nodulation, root development and phytohormone biosynthesis (Aung et al., 2017). Taking together, miR156 can improve drought or heat stress tolerance in alfalfa, at least partially by silencing *SPL13* (Feyissa et al., 2019; Matthews et al., 2019).

RNA-Seq analysis was utilized in the transcriptome profiling of alfalfa in order to study the molecular mechanisms underlying frost (Song et al., 2016), salinity (Postnikova et al., 2013; An et al., 2016; Lei et al., 2018), drought (Arshad et al., 2018), resistance to aluminum (Liu W. et al., 2017), lead (Xu et al., 2017) and waterlogging (Zeng et al., 2019), or fall dormancy (Zhang S. et al., 2015). For example, genes encoding membrane proteins, and proteins of hormonal signal transduction, and ubiquitin-mediated proteolysis pathways contribute to the freezing adaptation mechanisms in alfalfa (Song et al., 2016). Using high-throughput sequencing technology, Postnikova et al. (2013) have demonstrated that salinity stress affects a variety of alfalfa genes. Among the most affected ones were genes of known function, such as *DIHYDROFLAVONOL REDUCTASE* (*DFR)*, transcription factor *MYB59*, *SUGAR TRANSPORTER ERD6-like 16* (*ERD* – *EARLY RESPONSE TO DEHYDRATION*), and *INOSITOL-145-TRISPHOSPHATE 5-PHOSPHATASE* (*IP5P2*). This study revealed that 86 transcription factors responded to salinity stress; among them are those belonging to GRAS, ARR, JUMONJI, and MYB families that were preferentially upregulated in the tolerant alfalfa cultivar (Postnikova et al., 2013). Alfalfa fall dormancy is determined by genes involved in auxin (e.g., *AUXIN-INDUCED PROTEIN 5NG4)* and ethylene signaling (ethylene responsive TF *RAP2-11)* and carbohydrate transport *(ERD6-LIKE PROTEIN*; Zhang S. et al., 2015). Genes encoding *BETA-AMYLASE*, *ETHYLENE RESPONSE FACTOR (ERF)*, *CALCINEURIN B-LIKE (CBL) INTERACTING PROTEIN KINASES (CIPKs)*, *GLUTATHIONE PEROXIDASE (GPX)*, and *GLUTATHIONE S-TRANSFERASE (GST)* are among those important for waterlogging stress resistance in alfalfa (Zeng et al., 2019).

Plant damage caused by saline stress is usually divided into three categories: high pH damage, osmotic shock, and toxic cation stress. Nutrient solution pH variation significantly affected growth of alfalfa seedlings with the optimal pH values in the range between 5.0 and 6.0, as estimated by length and fresh weight of roots, hypocotyls, epicotyls, first leaf petioles, and leaf blades (Köpp et al., 2011). Alfalfa is a saline-alkaline stress-tolerant species (Zhu, 2001; Wong et al., 2006; Gong et al., 2014; An et al., 2016). An et al. (2016) performed transcriptomic analysis of whole alfalfa seedlings treated with saline-alkaline solutions using ion torrent sequencing technology to study changes in the gene expression pattern. This method detects hydrogen ions that are released during DNA polymerization. DEG profiles were obtained and annotated using two methods. Firstly, generated reads were mapped to barrel medic, which has a sequence that is highly homologous to alfalfa. Secondly, functional annotations of assembled unigenes were performed using BLASTX search against the Swiss-Prot databases of barrel medic, thale cress, and soybean. Gene ontology analysis revealed 14 highly enriched pathways. Specific responses of peroxidases, the expression level of *RUBISCO*, and flavonoids indicated antioxidant capacity as one of the main mechanisms behind the saline-alkaline stress tolerance in alfalfa (An et al., 2016). Another study provided a comprehensive transcriptome analysis of alfalfa roots under prolonged ABA treatment (Luo et al., 2019a). Sequences were assembled for many isoforms and were analyzed for their potential role. Differentially expressed isoforms (DEIs) regulated by ABA were mainly involved in transcriptional regulation, plant immunity, plant hormone signal transduction, and anti-oxidative defense.

Nevertheless, these studies were mainly focused on genotype-specific stress mechanisms. Functional and structural genomics studies are fundamental for the understanding of plant biology. Access to high-quality genome and transcriptome sequences is important to perform studies of this kind. Recently, the third-generation sequencing technology PacBio RSII has emerged as a unique method for constructing full-length transcripts (Dong et al., 2015; Nakano et al., 2017). PacBio RSII is an ideal tool for whole genome sequencing, targeted sequencing, RNA-Seq, and epigenetic characterization. This technique allows the sequencing of single DNA molecules in real-time (SMRT) without amplification by PCR (Dong et al., 2015). Using PacBio RSII, Luo et al. (2019b) studied salt stress as a major environmental factor that impacts alfalfa development and production (Zhang S. et al., 2015). They have constructed the first full-length transcriptome database of alfalfa root tips treated with mannitol (a non-ionic osmotic stress) and NaCl (an ionic osmotic stress), which provided evidence that the response to salinity stress includes both osmotic and ionic components. They have found 8,016 mannitol-regulated DEGs and 8,861 NaCl-regulated DEGs. These DEGs are involved in signal transduction, transcriptional regulation, anti-oxidative defense, and signal perceptions (Luo et al., 2019b).

### Resistance to Biotic Stress

Biotic stress also considerably affects alfalfa growth and yield. Current methods of plant protection focus mostly on the elimination of pathogenic organisms using pesticides (Shafique et al., 2014). However, the improvement of plant resistance against such pathogens seems like a more beneficial alternative, since it might be more effective and more environmentally friendly (Kudapa et al., 2013; Varshney and Kudapa, 2013). It is expected that climatic changes are linked to the spread of diseases and emergence of new ones and can raise the threat of parasites and pests (Kudapa et al., 2013; Shafique et al., 2014). Therefore, disease-resilient plants could provide higher production and yield, reflecting the importance of genetically engineering specific genes (de Zélicourt et al., 2011).

Disease resistance mechanisms in plants after encountering a pathogen have been well-described (Roumen, 1994; Zipfel, 2014; Rubiales et al., 2015). Plant infection is facilitated by effector molecules produced by pathogens, which can overcome the first line of plant defense, which is the pathogen−associated molecular pattern (PAMP) triggered immunity; subsequently, plant resistance is suppressed. On the other hand, specific plant resistance (R) proteins have been evolutionarily developed and can provide protection against specific pathogen effectors (Jones and Dangl, 2006; Singer et al., 2018). Nowadays, genes encoding R proteins are widely manipulated for introducing plant resistance to a specific pathogen (Rubiales et al., 2015).

Generally, the most frequently occurring pathogens are bacteria and fungi belonging to Ascomycetes and Basidiomycetes; these obtain nutrients by attacking various parts of the plant body (Shafique et al., 2014). Considerable declines in alfalfa production have been observed mostly due to root infections leading to wilting caused by the bacterium *Clavibacter michiganensis*, fungi *Fusarium oxysporum* and *Verticillium alfalfae*, and microscopic fungus *Phytophthora medicaginis*, or due to leaves infected by *Colletotrichum trifolii* (Nutter et al., 2002; Singer et al., 2018). Alfalfa varieties resistant to these diseases have been obtained by common breeding methods over decades (Toth and Bakheit, 1983; Elgin et al., 1988; Pratt and Rowe, 2002). However, it may not be enough to cover the world demand for crop yields, considering the influence of a retrogressive living environment. Because of alfalfa autopolyploidy and its out-crossing nature (Zhang T. et al., 2015; Yu et al., 2017), the comprehension of molecular and genetic mechanisms during pathogenesis leading to the introduction of specific resistance can be a demanding task. For this reason, barrel medic is widely used for such purposes. Different transcriptomic methods (Gao et al., 2012; Van et al., 2013; Le Nguyen et al., 2018; Scheben et al., 2019) were used to identify barrel medic loci correlated with QTLs, providing resistance to diseases caused by fungi such as *Uromyces striatus* and *Erysiphe pisi* (Bustos-Sanmamed et al., 2013).

*C. trifolii* is an agent of a highly destructive and prevalent foliar disease, anthracnose (Annicchiarico et al., 2015), which can cause up to 30% decrease in alfalfa yield (Yang et al., 2008). Recognition of this pathogen and induction of response in alfalfa are understudied and need further characterization by cloning techniques. Nevertheless, Yang et al. (2008) found out that overexpression of the gene for intracellular R protein, *RCT1* encoding TIR-NBS-LRR (TOLL/INTERLEUKIN-1 RECEPTOR NUCLEOTIDE BINDING SITE LEUCINE-RICH REPEAT) from barrel medic, ensured anthracnose resistance in alfalfa. Mackie et al. (2007) and Tesfaye et al. (2007) identified tetrasomic dominant *ANTHOCYANIN* genes *AN1* and *AN2* regulating resistance against *C. trifolii* (Elgin and Ostazeski, 1985). Mackie et al. (2007) mapped locations of QTLs for *C. trifolii* traits 1, 2, and 4 in autotetraploid alfalfa clone W126, which is resistant to this pest. Interactions between particular QTLs and phenotypic variations for three *C. trifolii* traits have been described. Obtained markers may be usable in alfalfa breeding for introducing multiple sources of resistance. Although genes for a specific resistance have been identified, new pathotypes of *C. trifolii* are still being discovered; therefore, the generation of new, long-lasting resistant plants is more difficult (Shafique et al., 2014).

Using the suppression subtractive hybridization library, Bustos-Sanmamed et al. (2013) proved the importance of pathogenesis-related (PR) proteins of group 10, as well as proteins engaged in ABA signaling for resistance against harmful fungi, e.g., *Aphanomyces enteiches*. Bahramnejad et al. (2010) designated and isolated the *MsPR10.1A* gene in alfalfa based on its homology to *PR10* genes from other Fabaceae plants, e.g., *Lupinus luteus* (Zhang, 2004). Expression levels of *MsPR10.1A* under different conditions such as ABA treatment, heat shock, wounding, and pathogen attack, were compared with the expression levels of a previously described gene, *PPRG2* (termed as *MsPR10.1B;* Borsics and Lados, 2002). Bahramnejad et al. (2010) observed faster induction of *MsPR10.1A* gene expression than that of *MsPR10.1B* gene after ABA and ethylene treatment, and after application of the pathogenic bacterium *Xanthomonas campestris*. However, inoculation of alfalfa leaves with compatible *X. campestris* led to markedly higher expression of both genes. On the other hand, gene *AAB41557* from the alfalfa *PR10* group did not respond to *X. campestris* inoculation (Esnault et al., 1993). Generally, most examples regarding *PR10* induction due to bacterial inoculation involve incompatible bacteria, such as activation of alfalfa genes *AAB41557* (Esnault et al., 1993) and *MsPR10.1B* (Borsics and Lados, 2002) after *Pseudomonas syringae pv. pisi* inoculation. The promoter of *YPR-10* (of the *RIBONUCLEASE-LIKE PR PROTEIN-10* gene) from *G. max* fused with *GUS* showed activity in the vasculature of *Nicotiana benthamiana* leaves after transient transformation (Walter et al., 1996). Moreover, Bahramnejad et al. (2010) suggested the importance of *MsPR10.1A* promoter expression in the leaf vasculature, resulting in resistance against diseases. *MsPR10.1A* and *MsPR10.1B* promoters have many similar functions in stress responses, but notable differences were found in their reactions to wounding. Thus, promoters of *PR10* genes may be potentially used in biotechnological applications for directing transgene expression in proper tissues.

Plant defense peptides are composed of five main groups: proteases, α-amylase inhibitors, lectins, chitinases, and polyphenol oxidases (Fürstenberg-Hägg et al., 2013). Singer et al. (2018) summarized several genes for the biosynthesis of substances with anti-pathogen effect, such as *AGLUL* encoding β-1,3-glucanase (Masoud et al., 1996), *IOMT* – Isoflavone-*O*-methyltransferase (He and Dixon, 2000), *LF* – encoding lactoferrin (Stefanova et al., 2013) and *RS* – encoding resveratrol synthase (Hipskind and Paiva, 2000). Highly effective protectants, such as protease inhibitors, naturally occur in plants, and they can inhibit proteolytic enzymes in the digestive system of insects or nematodes. Consequently, plant material is not digestible, leading to pathogen starvation and removal from the plant. Inhibitors of cysteine proteases called phytocystatins were identified in many plants, showing potential in conferring resistance against pathogens. Rice *ORYZACYSTATIN-I* (*OC-I*) and *ORYZACYSTATIN II* (*OC-II*) genes driven by a potato wound-inducible promoter (*Protease inhibitor II*, *PinII*) were transferred to alfalfa attacked by root lesion nematode and leaf beetle. Such transgenic plants revealed a reduction in the *Pratylenchus penetrans* population and enhanced mortality of *Phytodecta fornicata* larvae (Ninković et al., 1995; Samac and Smigocki, 2003).

Tesfaye et al. (2005) generated alfalfa plants that secreted a fungal endochitinase (ECH42). These transgenic plants showed up to 25.7 times increased chitinase activity in vegetative organs and root exudates. Such secreted endochitinases not only retained the lytic activity against glycol chitin, but also showed antifungal activity by the inhibition of spore germination of two fungal pathogens, namely, *Phoma medicaginis* and *C. trifolii* (Tesfaye et al., 2005).

Based on the expression distribution of *SNAKIN* gene *StSN1* in *Solanum tuberosum*, Segura et al. (1999) hypothesized *SN1* as a component of constitutive defense barriers in reproductive and storage plant organs. *StSN2* is induced locally after wounding and pathogen attack; accordingly, it could play an important role in constitutive and inducible defense barriers (Kovalskaya and Hammond, 2009; Guzman-Rodriguez et al., 2013). Next, García et al. (2014) proposed SNAKIN proteins as antimicrobial compounds in plant innate immunity. Indeed, alfalfa transgenic plants carrying *SNAKIN-1* (*MsSN1*) under the control of a constitutive promoter showed improved tolerance against pathogenic fungi. Three independent transgenic lines carrying the *CaMV35S:MsSN1* construct showed significantly lower amounts of infected leaves than wild type plants when treated by *C. trifolii* and with the oomycete *P. medicaginis* (García et al., 2014).

Finally, it is worth mentioning that the genetic transformation of alfalfa with *Bacillus thuringiensis* gene *Cry1C* coding for δ-endotoxin has also been shown to be an effective protective strategy. After transformation, alfalfa was more resistant to *Nemapogon granellus* and *Spodoptera exigua* (Strizhov et al., 1996).

Transcriptomic studies contributed to the knowledge of alfalfa resistance to aphids, strips, and nematodes. Aphids are major insect pests causing a significant decrease of alfalfa yield. Tu et al. (2018b) performed a transcriptomic analysis of two alfalfa cultivars differing in aphid resistance. Genes involved in salicylic acid biosynthesis represented an important defense mechanism in both cultivars. The alfalfa resistance against aphids was mainly determined by induction of genes involved in linoleic acid synthesis important for jasmonic acid and flavonoid biosynthesis (Tu et al., 2018b). Genes participating in jasmonic acid biosynthesis, such as *LIPOXYGENASE, SERINE PROTEINASE INHIBITOR*, and *SEED LINOLEATE 9S-LIPOXYGENASE* were also important for alfalfa resistance to strips infestation. Moreover, genes involved in fatty acid degradation, chloroalkane and chloroalkene degradation, beta-alanine and phenylalanine metabolism and flavonoid biosynthesis also contributed to this resistance (Tu et al., 2018a). Another comparative transcriptomic analysis aimed to screen for genes determining alfalfa resistance to root-knot nematode *Meloidogyne incognita* (Postnikova et al., 2015). *LRR AND NB-ARC DOMAIN DISEASE RESISTANCE PROTEIN* (Medtr3g023030.1), *RECEPTOR-LIKE PROTEIN* (Medtr5g087320.1) and *DISEASE RESISTANCE PROTEIN* (TIR-NBS-LRR class, Medtr0277s0020.3) were up-regulated in the resistant cultivar, while susceptible one showed their down-regulation (Postnikova et al., 2015).

From the biotechnological point of view, ideal alfalfa cultivars should have better nutritional quality, enhanced biomass production and yield, and better resistance to biotic and abiotic stress. All such traits mentioned should be sustainable over a long period of time. Several experimental studies have been conducted to improve alfalfa, but detailed characterization and relationships between desired traits need further genetic and molecular research.

## Proteomics and Metabolomics

Owing to its beneficial agronomical traits, alfalfa has been attracting substantial interest in the fields of proteomics and metabolomics during the past two decades. A strong effort was invested in the discovery of new proteins and metabolites involved in alfalfa development and abiotic stress response. In this section, we attempt to summarize the recent achievements of current alfalfa proteomic and metabolomic research. We also aim to highlight the relevance of these investigations for putative biotechnological applications.

### Nitrogen and Carbon Metabolism in Alfalfa From a Proteomic Perspective

Proteomics and metabolomics have a remarkable capability to examine the balance between carbon and nitrogen metabolism under stress conditions in alfalfa during interactions with nitrogen-fixing bacteria (Aranjuelo et al., 2011, 2013). Water stress limits nitrogen fixation in nodules by the reduction of nitrogenase activity (Carter and Sheaffer, 1983; Aranjuelo et al., 2011) and Rubisco availability in leaves (Aranjuelo et al., 2005, 2011). The latter likely occurs due to Rubisco-enhanced proteolysis and lower abundance of RUBISCO ACTIVASE. Water stress also affected ammonia assimilation into amino acids, as evidenced by the upregulation of glutamine synthetase and decreased levels of glutamic acid and asparagine in leaves. The effects of water stress were followed by elevated photorespiration (exemplified by increased abundances of photorespiratory enzymes), lower demand for carbohydrates, and accumulation of soluble sugars. In nodules, water deprivation caused the attenuation of respiration, leading to CO_2_ recycling by PHOSPHOENOLPYRUVATE CARBOXYLASE. This likely occurred in order to support carbon skeletons for amino acid biosynthesis. The reduced respiration may also be a consequence of increased demand for compounds with osmoregulation capacity such as glycerol (Aranjuelo et al., 2013). The dynamic behavior of ammonia assimilation seems to be important for abiotic stress tolerance. It is likely that nitrogen is relocated from glutamic acid and asparagine, which are the main nitrogen sources in control conditions, to proline under stress conditions. Thus, proline might be an alternative nitrogen source under osmotic stress, and it seems that alfalfa may easily switch between proline biosynthesis and degradation (Zhang and Shi, 2018). Abiotic stresses caused accumulation of enzymes of nitrogen assimilation, such as GLUTAMINE SYNTHETASE and FERREDOXIN-DEPENDENT GLUTAMATE SYNTHASE (Rahman et al., 2016) as well as GLUTAMATE DEHYDROGENASE (Dai et al., 2017). Remarkably, heat stress positively affected the abundance of ASPARTATE AMINOTRANSFERASE and GLUTAMINE SYNTHETASE, indicating an enhancement of nitrogen metabolism (Li W. et al., 2013).

Clearly, Rubisco availability and homeostasis between carbon and nitrogen metabolism is crucial for plant performance under unfavorable environmental conditions. For this reason, the proteins regulating C and N metabolism, as well as stress related proteins (Table 1), appear to be prospective candidates for the biotechnological improvement of alfalfa.

**TABLE 1:** Overview of proteins and metabolites important for biotechnological improvement of alfalfa as revealed by proteomic and metabolomic studies.

Treatment, stress, condition	Sample	Methodological approach	Proteins and metabolites of biotechnological importance	References
Seed germination and osmopriming	Seeds	2-D gel electrophoresis (nano-LC MS/MS)	**Carbohydrate metabolism:** UDP glucose pyrophosphorylase **Protein destination and storage:** HSP70 and HSP20, GroEL-like chaperone, ATPase, vicilin, protein disulfide-isomerase precursor **Stress response:** annexin, peroxiredoxins, manganese superoxide dismutase, glyoxalase, lipoxygenase, glutathione *S*-transferase, thioredoxin	Yacoubi et al. (2011)
			**Proteolysis:** peptidase T1A, proteasome beta subunit, peptidase A1 pepsin	
Osmoprimed seeds germinating under salt stress	Seeds	2-D gel electrophoresis (nano-LC MS/MS)	**Small HSPs:** 18.2 kDa class I HSP **Methionine synthesis:** methionine synthase, cysteine synthase **Dehydration defense:** LEA proteins, PM22 **Others:** annexin, RNA-binding protein, heme oxygenase, glutathione *S*-transferase 9	Yacoubi et al. (2013)
PEG-induced osmotic stress	Roots of varieties contrasting in drought tolerance	iTRAQ (strong cation exchange fractionation and LC MS/MS)	**Stress and defense:** glutathione *S*-transferases, disease resistance response protein, epoxide hydrolase, chitinase, reticuline oxidase-like protein, low-temperature-induced 65 kDa protein, aldo/keto reductase, pirin-like plant protein, glucan endo-1,3-beta-glucosidase **Protein metabolism:** HSPs, lysine-ketoglutarate reductase/saccharopine dehydrogenase, phosphatidylethanolamine-binding protein, homoglutathione synthetase	Zhang and Shi (2018)
			**Signal transduction:** monooxygenases, cysteine-rich RLK (receptor-like kinase) protein, 12-oxophytodienoate reductase	
			**Cell wall:** beta xylosidase, xyloglucan-specific endoglucanase inhibitor protein, expansin-B1-like protein	
Salt stress	Roots of two cultivars contrasting in salt resistance	2-D gel electrophoresis (MALDI TOF/TOF)	**Oxidative stress:** peroxidase, peroxiredoxin **Protein folding:** protein disulfide isomerase **Metabolism:** NAD synthetase, UTP-glucose 1 phosphate uridylyltransferase **Fatty acid metabolism:** biotin carboxylase 3	Rahman et al. (2015)
			**Membrane transport:** V-ATPase	
Salt and drought stress	Seedlings	2-D gel electrophoresis (MALDI TOF-MS/MS)	**Salt stress:** caffeoyl-CoA 3-*O*-methyltransferase, peroxiredoxin, ubiquitin-conjugating enzyme, UV excision repair protein rad23, glutathione peroxidase	Ma et al. (2017)
			**Drought stress:** ubiquitin-conjugating enzyme, putative alcohol dehydrogenase, chaperonin 10	
Drought stress	Leaves of plants inoculated by *S. meliloti*	Proteomics: 2-D gel electrophoresis (LCMS/MS analysis) Metabolomics: GC TOF-MS	**Rubisco availability and regeneration:** rubisco activase, sedoheptulose-1,7-bisphosphatase, ribulose-phosphate 3-epimerase and phosphoribulokinase **Nitrogen metabolism:** glutamine synthetase	Aranjuelo et al. (2011)
			**Stress and defense response:** superoxide dismutase, dehydroascorbate reductase, 2-cys peroxiredoxin-like protein, 14-3-3-like protein	
			**Osmoprotectant metabolites:** proline, pinitol	
Drought stress	Nodules, roots, leaves	Proteomics: 2-D gel electrophoresis (LCMS/MS analysis) Metabolomics: GC TOF-MS	**Nodule proteome:** alpha 1,4-glucan protein synthase, lipoxygenase, PEP-carboxylase **Nodule N containing metabolites:** glutamine, asparagine **Nodule osmoprotectant metabolites:** glycerol, galactinol, myo-inositol, proline, sucrose, raffinose, fumaric acid and malate **Nodule metabolites with antioxidant capacity:** ascorbate, threonate	Aranjuelo et al. (2013)
Water deficit stress	Roots	2-D gel electrophoresis (MALDI TOF)	**Nitrogen metabolism:** glutamine synthetase, ferredoxin-dependent glutamate synthase	Rahman et al. (2016)
			**ABA biosynthesis:** 9-*cis*-epoxycarotenoid dioxygenase	
			**Stress response and oxidative stress:** ascorbate peroxidase, peroxiredoxin, calreticulin, stress-induced phosphoprotein, annexin	
			**Transcription:** basic helix-loop-helix (bHLH) transcription factor, agamous-like 65 **Other functions:** inward-rectifying potassium channel, auxin-independent growth promoter	
Heat stress	Leaves	2-D gel electrophoresis (MALDI TOF/TOF)	**Rubisco availability:** Rubisco activase isoforms **Nitrogen metabolism:** aspartate aminotransferase and glutamine synthetase	Li W. et al. (2013)
			**Protein synthesis and processing:** peptidyl-prolyl *cis*–*trans* isomerases, protein disulfide isomerase-like protein precursor, porin, proteasome subunit β type, eukaryotic translation initiation factor 3 subunit I, BiP isoform A/glycine max, cysteine proteinase, outer plastidial membrane protein porin	
			**Intracellular traffic, cell structure:** protein TOC75, translocon Tic40, profilin	
			**Defense response:** 17 kDa HSP, 18.2 kDa class I HSP, 20 kDa chaperonin, HSP23, HSP70, thaumatin-like protein, ubiquitin, ascorbate peroxidases, glucan endo-1,3-beta-glucosidase	
Cold acclimation	Leaves of cultivars tolerant or sensitive to freezing	2-D gel electrophoresis (MALDI TOF/TOF)	**Oxidative stress:** monodehydroascorbate reductase, glutathione peroxidase, peptide methionine sulfoxide reductases A3, thioredoxin-like protein CDSP32, 2-cys peroxiredoxin BAS1-like	Chen et al. (2015)
			**Methionine biosynthesis:** 5-methyltetrahydropteroyltriglutamate-homocysteine methyltransferase	
			**Lignin and terpenoid biosynthesis:** cinnamoyl-CoA reductase, 1-deoxy-D-xylulose 5-phosphate reductoisomerase	
			**Photosynthesis and Rubisco availability:** Rubisco large subunit-binding protein subunit beta, Rubisco activase B, chlorophyll A/B binding protein, oxygen-evolving enhancer protein 1, cytochrome b6-f complex iron-sulfur subunit	
			**Protein folding and disassembling:** chaperone protein ClpC, GTPase, peptidyl-prolyl *cis*–*trans* isomerase CYP20-3	
Cadmium stress	Cell walls and soluble proteins from stems	2-D DIGE (MALDI TOF/TOF)	**Cell wall modification:** sucrose synthase, pectinesterase/pectinesterase inhibitor, polygalacturonase non-catalytic protein, polygalacturonase-inhibiting protein 1, b-xylosidase/alpha-L-arabinofuranosidase, trichome birefringence-like protein, xyloglucan endotransglucosylase/hydrolase family protein, dirigent protein 21-like	Gutsch et al. (2018a)
			**Defense:** chitinase (Class Ib)/hevein, chitinase, class I chitinase, disease resistance response protein, pathogenesis-related protein 1, pathogenesis-related thaumatin family protein, plant basic secretory protein (BSP) family protein, pre-hevein-like protein, stromal 70 kDa heat shock-related protein, CAP, cysteine-rich secretory protein, antigen 5	
			**Oxidation-reduction process:** anionic peroxidase swpb3 protein, class III peroxidase, peroxidase family protein, peroxidase1b, peroxidase2	
Cadmium stress	Stems (soluble and cell wall enriched proteins)	2-D DIGE (MALDI TOF/TOF)	**Cell wall modification:** pectinesterase/pectinesterase inhibitor, polygalacturonase non-catalytic protein, polygalacturonase-inhibiting protein 1 **Chloroplast protein degradation:** chloroplastic aspartyl protease isoforms **Cell wall:** class III peroxidase, lignin biosynthetic peroxidase, chitinases	Gutsch et al. (2018b)
Stem growth	Different regions of stems (apical, intermediate, and basal)	2-D gel electrophoresis (MALDI TOF/TOF)	**Chloroplast protein synthesis:** CSP41-b, EF-Tu, EF-G, Cpn 60, HSP70 **Lignin biosynthesis:** transketolase, enolase **Cytoplasmic protein synthesis:** eIF-5a, endoplasmic protein disulfide isomerase, HSP90, ribosomal protein P3-like	Printz et al. (2015)
			**Vesicular trafficking:** clathrin light chain	
			**Stress response:** peroxisomal membrane protein, monodehydroascorbate reductase, flavoprotein wrbA-like, Pprg2	
			**Sieve element development:** sieve element occlusion by forisomes 3	
Cadmium stress and hydrogen- rich water	Roots	iTRAQ (nano-LC MS/MS)	**Defense response:** mitogen-activated protein kinase, pathogenesis-related thaumatin family protein, pathogenesis-related protein bet V I family protein, disease-resistance response protein **Nitrogen metabolism:** glutamate dehydrogenase **Sulfur compound metabolic process:** cysteine synthase, ATP sulfurylase	Dai et al. (2017)
			**Secondary metabolism:** chalcone-flavonone isomerase family protein	
Waterlogging	Leaves of two cultivars contrasting in tolerance to waterlogging	iTRAQ (reverse-phase HPLC fractionation and LC-MS/MS)	**Cell wall and defense response:** acidic endochitinase, expansin-like B1, early nodulin-like protein 2, thaumatin-like protein, 1,4 alpha-glucan-branching enzyme 1, pathogenesis-related protein **Stress response:** glutathione *S*-transferase, protein C2-DOMAIN ABA-RELATED 9, aldo-keto reductase family 4 member C9, Fe superoxide dismutase 2, 1 aminocyclopropane-1-carboxylate oxidase homolog 5,	Zeng et al. (2019)
			**Proteolysis:** vacuolar-processing enzyme	
Different developmental stages (budding and mid-flowering)	Leaves	TMT labeling (nano-LC MS/MS)	**Metabolites:** D-mannose hemicellulose precursor (upregulated in mid flowering), L-phenylalanine, L-tyrosine, L-phenylalanine **Metabolism:** alpha glucosidase, alpha amylase **Cell wall modification:** UDP-glucuronic acid decarboxylase (xylan production), cinnamyl alcohol dehydrogenase (lignin biosynthesis)	Fan et al. (2018)
Fall dormancy	Terminal buds of fall dormant and non-fall dormant cultivars	iTRAQ (SCX fractionation, LC MS/MS)	**Nitrogen metabolism:** L-asparaginase **Auxin polar transport:** stilbene synthase family protein, monothiol glutaredoxin-S17 protein **Lignin biosynthesis:** cinnamyl alcohol dehydrogenase **Pyruvate metabolism and transport:** pyruvate carrier protein	Du et al. (2018)
			**Vitamin B1 metabolism:** thiazole biosynthetic enzyme	

### Proteins and Pathways Found by Proteomics as Promising Candidates for Alfalfa Abiotic Stress Resistance Improvement

Seed priming involves a complex array of physiological as well as molecular processes leading to an improved ability of plants to withstand adverse environment (Paparella et al., 2015). A gel-based proteomic approach was employed to investigate proteome remodeling during osmoprimed alfalfa seed germination. This process was accompanied by intense accumulation of storage proteins (such as vicilins), proteins involved in protein folding, UDP glucose and methionine biosynthesis, annexins, and antioxidant enzymes, compared to seeds that were not osmoprimed. Osmopriming was also followed by remarkable induction of stress-related proteins and proteasome components (Table 1) (Yacoubi et al., 2011). A follow-up article highlighted that osmopriming has remarkable consequences on the proteome of seeds germinating under saline conditions. An increased seed vigor associated with osmopriming was related to the accumulation of storage proteins, annexins and RNA-BINDING PROTEIN. The last one indicated the possible importance of posttranscriptional regulation in the seedlings exposed to salt stress. On the other hand, seeds without osmopriming accumulated HEAT SHOCK PROTEINS (HSP), LATE EMBRYOGENESIS ABUNDANT (LEA) PROTEINS, SEED MATURATION PROTEINS, GLUTATHIONE *S*-TRANSFERASE 9, and HEME OXIDASE (Table 1) (Yacoubi et al., 2013). These data indicate that the transient genetic modification of genes encoding the above-mentioned stress-related proteins (for instance, by expression under an inducible tissue-specific promoter), might be of biotechnological importance.

Tolerance of alfalfa to the polyethylene glycol (PEG)-induced osmotic stress was accompanied by enhanced carbohydrate metabolism and energy production. Stress-related proteins such as glutathione *S*-transferases and LEA proteins are also correlated with osmotic stress tolerance (Table 1) (Zhang and Shi, 2018), and both represent promising candidates for biotechnological applications. A similar study revealed that proteins involved in protein folding (DISULFIDE ISOMERASE), NAD production (NAD SYNTHASE), methylation (ADENOSINE KINASE, *S*-ADENOSYL-METHIONINE) and antioxidant defense (represented mainly by peroxidases), are candidates to determine alfalfa salt tolerance (Rahman et al., 2015). Overabundance of proteins involved in the enzymatic antioxidant defense was commonly associated with an increased tolerance of alfalfa not only to the salt, but also to the drought and osmotic stresses (Table 1) (Rahman et al., 2015; Long et al., 2018; Zhang and Shi, 2018). According to a proteomic study, water stress increased the abundance of AGAMOUS-LIKE 65 and bHLH TRANSCRIPTION FACTORS, while it reduced the abundance of JADE-1 and JADE-3, transcriptional regulators belonging to a PHD (plant homeodomain)-type zinc fingers family (Table 1) (Rahman et al., 2016). These intriguing findings of transcriptional factors involved in water stress deserve further biotechnological investigations. Genetic modifications of hormone biosynthesis belong also to promising biotechnological approaches, since water stress elevated the abundances of ABA (9-*CIS*-EPOXYCAROTENOID DIOXYGENASE) and auxin (AUXIN-INDEPENDENT GROWTH PROMOTER) biosynthetic proteins in alfalfa (Rahman et al., 2016). In this regard, local stress-induced changes in the turnover of auxin regulatory proteins could modify plant developmental processes, such as cell elongation, lateral roots emergence, transition from cell division to cell differentiation, enabling plants to rapidly adapt to adverse environmental conditions (Korver et al., 2018). On the other hand, drought stress caused some common but also distinct responses when compared to salt stress at the level of the alfalfa proteome. Interestingly, both stresses targeted proteasome complex and translation. Nevertheless, the proteasome complex exhibits different sensitivity to these stressors, since the abundance of 26S PROTEASOME REGULATORY SUBUNIT 6 was increased by drought but subsequently reduced by salt stress (Ma et al., 2017).

Comparative proteomic studies point out to obvious similarities between alfalfa and barrel medic in their response to environmental stimuli. Proteome-wide comparison of salt-tolerant alfalfa and salt-sensitive barrel medic indicated that both species are capable of keeping photosynthetic activity during salt stress. Only heat shock protein (gi357476131) was differentially regulated under salt stress in these two Medicago species. It was upregulated in alfalfa but downregulated in barrel medic (Long et al., 2016), indicating its potential biotechnological significance for salt tolerance. A proteomic analysis of these two species at the early post-germination stage showed an important role of antioxidant defense, cell wall metabolism, and jasmonic acid biosynthesis during response to salt (Long et al., 2018). Enhanced salt tolerance of alfalfa, compared to salt sensitive barrel medic, was reflected by higher numbers of differentially regulated proteins, also suggesting higher proteome plasticity (Long et al., 2016, 2018).

Differences in the composition of differentially abundant proteins between two alfalfa cultivars with contrasting freezing tolerance were reported after cold stress treatment (Chen et al., 2015). Freezing-tolerant cultivar exhibited higher abundances of Rubisco subunits as compared to the freezing susceptible one, but showed downregulation of proteins involved in methionine, lignin and terpenoid biosynthesis, and energy metabolism under cold stress (Chen et al., 2015). Heat stress caused an upregulation of proteins involved in energy production, signaling, and intracellular transport and defense, including chaperones, antioxidant enzymes and PR proteins (Li W. et al., 2013). Interestingly, only prolonged heat stress caused downregulation of Rubisco and photosynthetic enzyme activities. Lower abundance of photosynthetic proteins was associated with altered abundance of proteins involved in plastid protein import.

It is known that the external application of bioactive molecules such as hydrogen (H_2_; Jin et al., 2013) may remarkably increase plant survival rate under adverse environmental conditions. Proteomic elucidation of the beneficial effects of H_2_ on the alfalfa response to cadmium revealed that this is mainly determined by the modification of proteins involved in the cellular redox homeostasis. Among these proteins, enzymes involved in cysteine biosynthesis and CYSTEINE DESULFURYLASE are elevated by external H_2_. Cysteine is a precursor for GSH and hGSH, an important redox buffering compounds (Baldacci-Cresp et al., 2012; Diaz-Vivancos et al., 2015), hGSH is specifically produced in species of Fabaceae family including alfalfa, in higher rate compared to GSH, having important role in nodulation (Klapheck, 1988; Matamoros et al., 1999; Frendo et al., 2005; Baldacci-Cresp et al., 2012; Pasternak et al., 2014). Similarly, the abundance of CuZn SUPEROXIDE DISMUTASE (SOD) increased along with a positive effect of external H_2_ treatment on alfalfa Cd tolerance. Gaseous H_2_ also enhances the abundance of defense related proteins such as PATHOGENESIS-RELATED PROTEIN BET V I FAMILY PROTEIN and PATHOGENESIS-RELATED THAUMATIN FAMILY PROTEIN (Dai et al., 2017). Such induction of defense related proteins, including chitinases and enzymes involved in cell wall modification, was also observed in alfalfa stems and leaves exposed to long-term Cd stress (Gutsch et al., 2018a, b). Remarkably, chitinases are also employed in the alfalfa response to osmotic stress and waterlogging (Table 1) (Zhang and Shi, 2018; Zeng et al., 2019). This implies that genetic modification of cell walls might improve alfalfa tolerance to multiple stresses.

### Proteins Implicated in Development-Associated Agronomical Traits

Proteomics has also been proven as valuable for the evaluation of metabolic activities during alfalfa stem development. The apical region characterized by fiber development showed an overabundance of proteins involved in chloroplast protein synthesis and carbon fixation. The mature stem part possessed a pool of proteins involved in redox homeostasis (Printz et al., 2015). Moreover, the stem is an organ highly sensitive to perturbations of mineral nutrition. This was highlighted by recent proteomic studies reporting that copper availability greatly influenced the abundance of proteins involved in cell wall biogenesis, and in pectin and lignin biosynthesis (Printz et al., 2016). Thus, mineral homeostasis seems to be a crucial factor affecting alfalfa stem growth and rigidity, and also eventually affecting drought tolerance and pathogen resistance.

Flowering represents a critical developmental stage in alfalfa, mainly in terms of seed yield and quality. Pollination and post-pollination processes in alfalfa are linked to altered homeostasis of stress-related proteins such as DUAL SPECIFICITY KINASE SPLA-LIKE PROTEIN, NADPH: QUINONE OXIDOREDUCTASE-LIKE PROTEIN, and CARBONIC ANHYDRASE (Chen et al., 2016). Moreover, PROTEIN DISULFIDE ISOMERASE-LIKE PROTEIN, ASCORBATE PEROXIDASE, GLUTAREDOXIN, and PEROXIREDOXINS also showed fluctuations in their abundances. In addition, metabolic activity was enhanced during pollination and declined afterward.

Fall dormancy is a crucial phenomenon influencing alfalfa performance in autumn, but also during the following season. Based on a comparative proteomic study of terminal buds isolated from two alfalfa cultivars with contrasting fall dormancy, several new proteins were discovered as important for this physiological process (Du et al., 2018). It was suggested that lower abundance of L-ASPARAGINASE and CINNAMYL ALCOHOL DEHYDROGENASES may contribute to fall dormancy. In addition, CHALCONE AND STILBENE SYNTHASE FAMILY PROTEIN (a protein involved in flavonoid biosynthesis) and GLUTAREDOXIN S17 seemed to be important for shoot apical meristem maintenance. Both proteins also have a role in polar auxin transport (Table 1) (Du et al., 2018).

Finally, the nutritional value of alfalfa depends on the developmental stage. Cutting of alfalfa in later developmental stages, such as in full flowering, leads to increased fiber and decreased protein content in the biomass (Fan et al., 2018). Combined proteomic and metabolomic analyses underpinned this finding and showed changes in amino acid composition. These unfeasible nutritional changes are accompanied by increased hemicellulose content, due to the accumulation of D-mannose and higher abundance of ALPHA GLUCOSIDASE, ALPHA AMYLASE, and UDP-GLUCURONIC ACID DECARBOXYLASE, as well as lignin, due to the higher levels of lignin precursors and proteins involved in lignin biosynthesis (Table 1) (Fan et al., 2018).

## Gene Editing Using Talen and CRISPR/Cas Technologies

The process of gene editing is based on sequence-specific nucleases (SSNs) creating *in vivo* loci-specific DNA double-stranded breaks (DSBs) that are subsequently repaired. There are two main DNA repair systems: homology-directed repair (HDR), and the more efficient but less precise non-homologous end joining (NHEJ). NHEJ can result in the insertion or deletion (indel) of nucleotides and a frameshift mutation, which can consequently create a premature stop codon, thus rendering the gene non-functional and creating a genetic knockout. Gene targeting technologies include meganucleases, zinc finger nucleases (ZFNs), transcription activator-like effector nucleases (TAL effector nucleases or TALENs), and clustered regularly interspaced short palindromic repeat/CRISPR–associated protein 9 (CRISPR/Cas9). Among these, TALENs and CRISPR/Cas9 are the preferred SSNs for research purposes (Kanaar et al., 1998; Pastwa and Blasiak, 2003; Smith et al., 2006; Pâques and Duchateau, 2007; Hartlerode and Scully, 2009; Sander et al., 2011; Qi, 2015; Steinert et al., 2016; Malzahn et al., 2017; Shan et al., 2020).

The history of gene targeting technologies started in 1988 when the first gene-targeting experiment was performed on tobacco (*Nicotiana tabacum*) protoplasts (Paszkowski et al., 1988). Later, Puchta et al. (1993) discovered that gene-targeting efficiency can be improved by DSBs in plant cells. More than a decade later, ZFNs were adapted in tobacco and were used in a few plant species for trait improvement (Wright et al., 2005). Subsequently, TALENs were introduced into the group of plant genome editing technologies (Christian et al., 2010). Finally, CRISPR/Cas9 technology has been used in plants such as *Arabidopsis thaliana*, *N. benthamiana*, *Oryza sativa*, and *T. aestivum* (Li J. F. et al., 2013; Nekrasov et al., 2013; Shan et al., 2013, 2020).

### TALENs

TALENs are created by the fusion of DNA binding TALE repeats to the Fok1 nuclease domain. TALENs are less toxic and are easier to engineer than ZFNs. Each of these two platforms has unique limitations, and they are not routinely used in plants. The main advantages of TALENs over CRISPR are that they have less off-target effects due to their ∼30 bp target requirement, as well as their lack of PAM requirement, as unlike CRISPR, TALENs are able to target any sequence. On the other hand, TALENs have more disadvantages: an increased time and financial investment due to the difficulty in protein engineering, a highly variable efficiency for each construct, an inability to target methylated DNA, and the difficulties in engineering nickase (Christian et al., 2010; Li et al., 2011; Mahfouz et al., 2011; Miller et al., 2011; Malzahn et al., 2017; Chen et al., 2019). So far, a successful application of TALEN technology has not been published for either alfalfa or barrel medic. Nevertheless, TALENs have been used for the targeted mutagenesis of another legume, namely soybean (Haun et al., 2014; Demorest et al., 2016; Du et al., 2016; Curtin et al., 2018). The use of TALENs for the mutagenesis of higher plants was recently reviewed by Malzahn et al. (2017) and Khan et al. (2017).

### CRISPR/Cas9

In bacteria and archaea, CRISPR and Cas9 function together against invading phages, plasmids, and viruses in adaptive immune system by cleaving the invader’s nucleic acids. The first component is single guide RNA (sgRNA) that associates with a Cas9 protein a Cas9/sgRNA complex. The second component Cas9 belongs to the single-protein effectors of Class 2 CRISPR-Cas systems and is composed of two endonuclease domains, namely, the RuvC-like domain and the HNH, each cutting one strand of DNA. The CRISPR/Cas9 constituents can be transformed into plant cells by different strategies, including *Agrobacterium*-mediated delivery, gene gun (biolistic delivery), or using virus-based guide RNA (gRNA). Out of the primary SSN classes, CRISPR/Cas9 technology has been the most used and adopted in recent years (Barrangou et al., 2007; Marraffini and Sontheimer, 2008; Wiedenheft et al., 2012; Graham and Root, 2015; Schiml and Puchta, 2016; Makarova et al., 2017; Malzahn et al., 2017; Chen et al., 2019). The CRISPR/Cas system has the potential for numerous applications, such as fusing dCas9 (deactivated Cas9) with other proteins, which can be used for DNA imaging, epigenome editing, gene regulation, and genomic labeling (Chen et al., 2019). One of the main limitations of CRISPR/Cas9 technology might be the generation of undesired off-target effects. Nevertheless, whole-genome sequencing revealed very limited off-target effect mutations in *Arabidopsis* (Feng et al., 2013), rice (Zhang H. et al., 2014; Tang et al., 2018), and tomato (Nekrasov et al., 2017). Using software tools such as CRISPR-P (Liu H. et al., 2017) and CRISPRGE (Xie et al., 2017) can further decrease any potential off-target occurrence by designing highly specific guide RNAs. Finally, breeding processes may remove any off-target mutations that have negative effects and may keep positive or neutral off-target mutations (Mao et al., 2019).

### CRISPR/Cas9 in Alfalfa

CRISPR/Cas9 technology was very recently used for targeted mutagenesis in alfalfa. Selected *SQUAMOSA PROMOTER BINDING PROTEIN-LIKE 9* (*SPL9*) gene was successfully mutagenized and transgenic lines were pre-selected by using droplet digital PCR (ddPCR) for high-throughput screening of large populations. It was further confirmed by restriction enzyme digestion after PCR amplification and sequencing of sub-clones. Comparison of editing efficiency with available data on barrel medic showed lower efficiency in alfalfa, which might be related to its tetraploid genome possessing highly repeated clusters (Meng et al., 2017, 2019; Gao et al., 2018). Gao et al. (2018) concluded that CRISPR/Cas9-mediated modifications of tetraploid alfalfa genome have been successfully performed, but there is still a need to improve editing efficiency. Alfalfa plants with silenced *SPL9* had no visible phenotype so ddPCR-based estimation of concentration of the event per μl was a direct indicator of the genome editing rate. Sequencing analysis showed no off-target effects in the alfalfa genome and proved that the sgRNAs of *SPL9* were highly specific to the recognition site. In other legumes such as barrel medic, CRISPR/Cas9 technology has been used as well (Michno et al., 2015; Meng et al., 2017, 2019; Curtin et al., 2018; Wen et al., 2019; Yin et al., 2020). Recently, Meng et al. (2019) developed an optimized *Agrobacterium*-dependent CRISPR/Cas9 system and successfully edited an endogenous *PHYTOENE DESATURASE* (*MtPDS)* gene. CRISPR/Cas9 technology for the mutagenesis was also used in *L. japonicus* (Wang et al., 2016, 2019), and *G. max* (Cai et al., 2015; Jacobs et al., 2015; Li et al., 2015; Sun et al., 2015; Du et al., 2016; Tang et al., 2016; Curtin et al., 2018; Bao et al., 2019; Wang et al., 2020). Utilization of CRISPR/Cas9-based mutagenesis in several non-leguminous plant species, including data on delivery method, integration into the genome, and editing efficiency, has been reviewed recently (Belhaj et al., 2013; Jaganathan et al., 2018; Liu X. et al., 2019; Kuluev et al., 2019; Mao et al., 2019; Moradpour and Abdulah, 2020; Shan et al., 2020). Approaches such as transgene integration and gene stacking developed for diploid crop species (e.g., corn, cotton, soybean) might be less suitable for alfalfa due to its auto-tetraploid character (Kumar et al., 2018), but the CRISPR/Cas9 technology seems to work well.

## Phosphorylation-Dependent Post-Translational Modification by MAPKs

Multiple abiotic stress stimuli, such as wounding, cold, salinity, or drought, are perceived by plants through the activation of MAPKs (Šamajová et al., 2013b). Activated MAPKs phosphorylate, and thereby regulate, several intracellular targets including other protein kinases, cytoskeletal components, nuclear transcription factors, and proteins involved in vesicular trafficking (Komis et al., 2011; Šamajová et al., 2013a). In alfalfa, STRESS-INDUCED MAPK (SIMK), was identified as a salt- and elicitor- stress induced MAPK (Cardinale et al., 2002). SIMK in response to salt stress is specifically activated by upstream STRESS-INDUCED MAPKK (SIMKK; Kiegerl et al., 2000; Bekešová et al., 2015). SIMK is localized to nuclei and cytoplasm of root cells, while in developing root hairs it relocated from the nucleus to the growing tip (Šamaj et al., 2002). Moreover, stimulus-dependent activation and the subsequent subcellular relocation of both SIMK and its upstream SIMKK were induced by salt stress (Ovečka et al., 2014). Such activity-dependent and coordinated relocation of SIMK-SIMKK module from the nucleus to cytoplasm under salt stress were observed in alfalfa and thale cress. Transgenic thale cress plants stably producing SIMKK-YFP exhibited enhanced MITOGEN-ACTIVATED PROTEIN KINASE 3 (MPK3) and MITOGEN-ACTIVATED PROTEIN KINASE 6 (MPK6) activation and conferred altered sensitivity to salt stress. These data suggested that SIMKK may serve as a negative regulator of the salt stress response in alfalfa (Ovečka et al., 2014).

## Conclusion and Perspectives

Alfalfa is a perennial, cross-pollinated, autotetraploid (2*n* = 4*x* = 32) plant with genome size of 800–900 Mbp. It is often mentioned as the “queen of forages” due to the very high production potential as hay, silage or as a biofuel feedstock for ethanol production (Blondon et al., 1994). However, tetraploid nature made understanding and improving of alfalfa by traditional breeding methods rather challenging. Therefore, the use of modern biotechnological, omics and genetic engineering approaches for alfalfa improvement is highly actual and desirable task for crop researchers.

**FIGURE 1:**
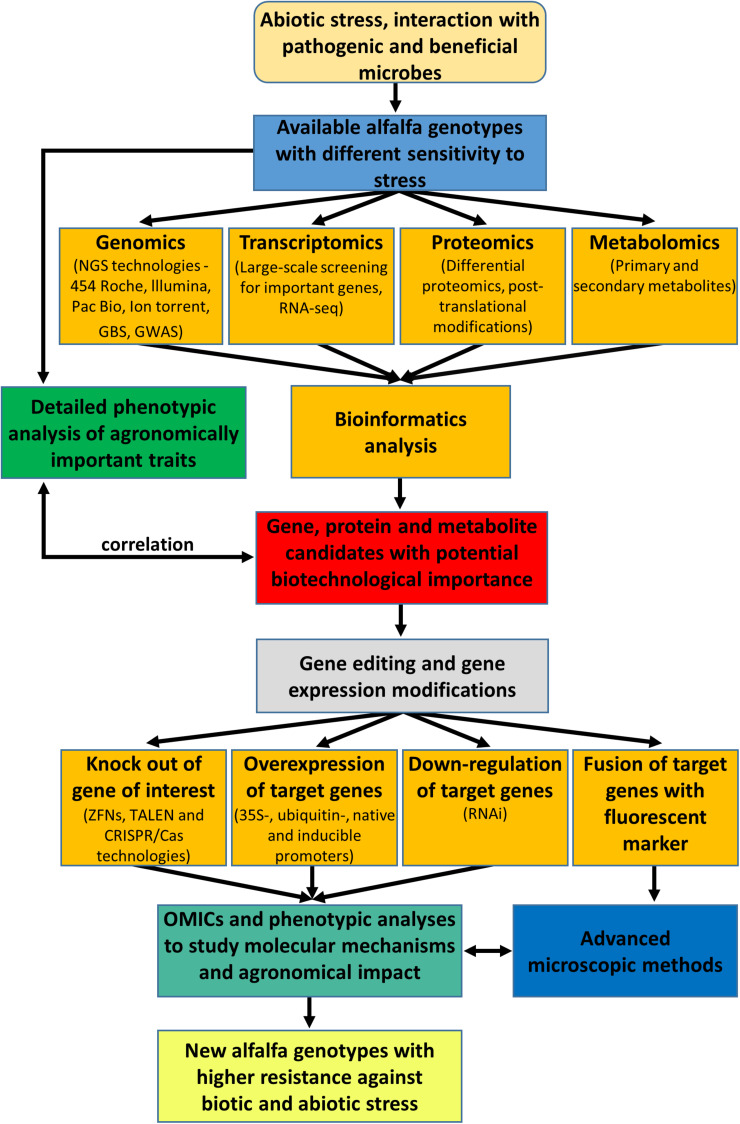
Overview and integration of omics and molecular genetics approaches aiming to improve agronomic traits and performance of alfalfa.

This review provides an overview of the biotechnological potential of alfalfa based on the integration of various omics and molecular tools as depicted in the Figure 1. Recent advances in high-throughput sequencing technology have opened another scientific boundary, and many species, including economically important crops, have been subjected to whole-genome sequencing by *de novo* assembly and resequencing. Several novel genes have been identified owing to whole-genome duplications and structural variations in chromosomes (Van et al., 2013). Since plant responses to stresses are often very specific, proteomic and transcriptomic approaches should be targeted to individual cell types and tissues at different developmental stages. Such approach was already reported for root hairs and root border cells of barrel medic (Breakspear et al., 2014; Watson et al., 2015). In this respect, the integration of fast-developing omics methods and bioinformatics into systems biology at the single cell level might bring new opportunities to improve plant stress tolerance (Libault et al., 2017).

Biotechnological approaches provide a great potential to increase crop production for the constantly growing global population. Introducing tolerance to environmental abiotic and biotic stresses is crucial for improving the productivity of crop legumes (Farooq et al., 2017). Extensive research conducted on alfalfa stress tolerance suggests that it is able to cope with abiotic stresses using general mechanisms such as antioxidant defense, protein folding, and cell wall remodeling. Research in the field of alfalfa biotechnology also aimed to identify genes involved in the energy production pathway or in enhancing environmental tolerance (Pennycooke et al., 2008; Aranjuelo et al., 2011; Mo et al., 2011). Scientists grew alfalfa plants under different conditions in order to analyze gene expression profiles and to identify crucial genes and proteins, as well as to understand global correlations between genes, proteins, and metabolites using omics approaches.

The potentials of these methods have only partially been exploited in alfalfa research. Continued research toward the development of alfalfa proteome studies (Komatsu and Ahsan, 2009) should permit the rapid comparison of alfalfa cultivars, mutants, and transgenic lines.

## Author Contributions

All authors listed have made a substantial, direct and intellectual contribution to the work, and approved it for publication. MH, PD, TT, MT, and IL drafted the review which was coordinated by OŠ, MO, and finally edited by JŠ.

## Conflict of Interest

The authors declare that the research was conducted in the absence of any commercial or financial relationships that could be construed as a potential conflict of interest.

## Footnotes

1 https://www.jcvi.org/research/medicago-truncatula-genome-database

2 phytozome.jgi.doe.gov/pz/portal.html#!info?alias=Org_Mtruncatula
